# The YYR (YY1- RKIP) Regulatory Axis in the pathogenesis of Cancer and Immune Evasion

**DOI:** 10.1186/s13046-025-03583-5

**Published:** 2025-12-02

**Authors:** William Ung, Benjamin Bonavida

**Affiliations:** Department of Microbiology, Immunology & Molecular Genetics, Jonsson Comprehensive Cancer Center, David Geffen School of Medicine, University of California at Los Angeles, Los Angeles, CA 90095 USA

**Keywords:** Cancer, Yin Yang1 (YY1), Raf kinase inhibitor protein (RKIP), YYR, Pathogenesis-therapeutic targeting

## Abstract

**Background:**

The transcription factor Yin Yang 1 (YY1) and the Raf kinase inhibitory protein (RKIP) represent two molecular entities with diametrically opposed roles in cancer biology. They are key modulators of multiple cellular processes, including apoptosis, metastasis, and cell survival. YY1 functions predominantly as an oncogenic driver, promoting tumorigenesis, epithelial-mesenchymal transition (EMT), immune evasion, and resistance to chemo-immuno-therapy. In contrast, RKIP acts as a metastasis suppressor and chemo-immuno-sensitizer, inhibiting critical oncogenic signaling pathways. The inverse correlation between high YY1 and low RKIP expressions has been observed across various malignancies (such as prostate cancer, melanoma, colorectal cancer, cervical cancer, hematologic malignancies, etc.), suggesting a tightly regulated molecular axis influencing tumor progression and therapeutic response. This review systematically examines the contrasting roles of YY1 and RKIP in cancer pathogenesis (e.g. cell proliferation and cell cycle, angiogenesis, immune cells infiltration and immunosuppressive TME, check point inhibitors, resistance to apoptosis, cell energetics, etc.). Based on their opposing activities, we propose the term YYR–the YY1–RKIP regulatory network– to explain the interplay. YYR captures the bidirectional and context-dependent nature of their relationship for understanding transcriptional programming, immune suppression, tumor aggressiveness, and therapeutic resistance in cancer.

**Conclusion:**

Understanding the dynamics of the YYR axis may offer new insights into prognostic markers and therapeutic strategies aimed at restoring tumor suppressor function and overcoming treatment resistance. Accordingly, we explore potential therapeutic strategies aimed at targeting YYR.

**Graphical Abstract:**

This figure represents the opposing activities mediated by the tumorigenic oncogene YY1 and the tumoricidal tumor suppressor RKIP.

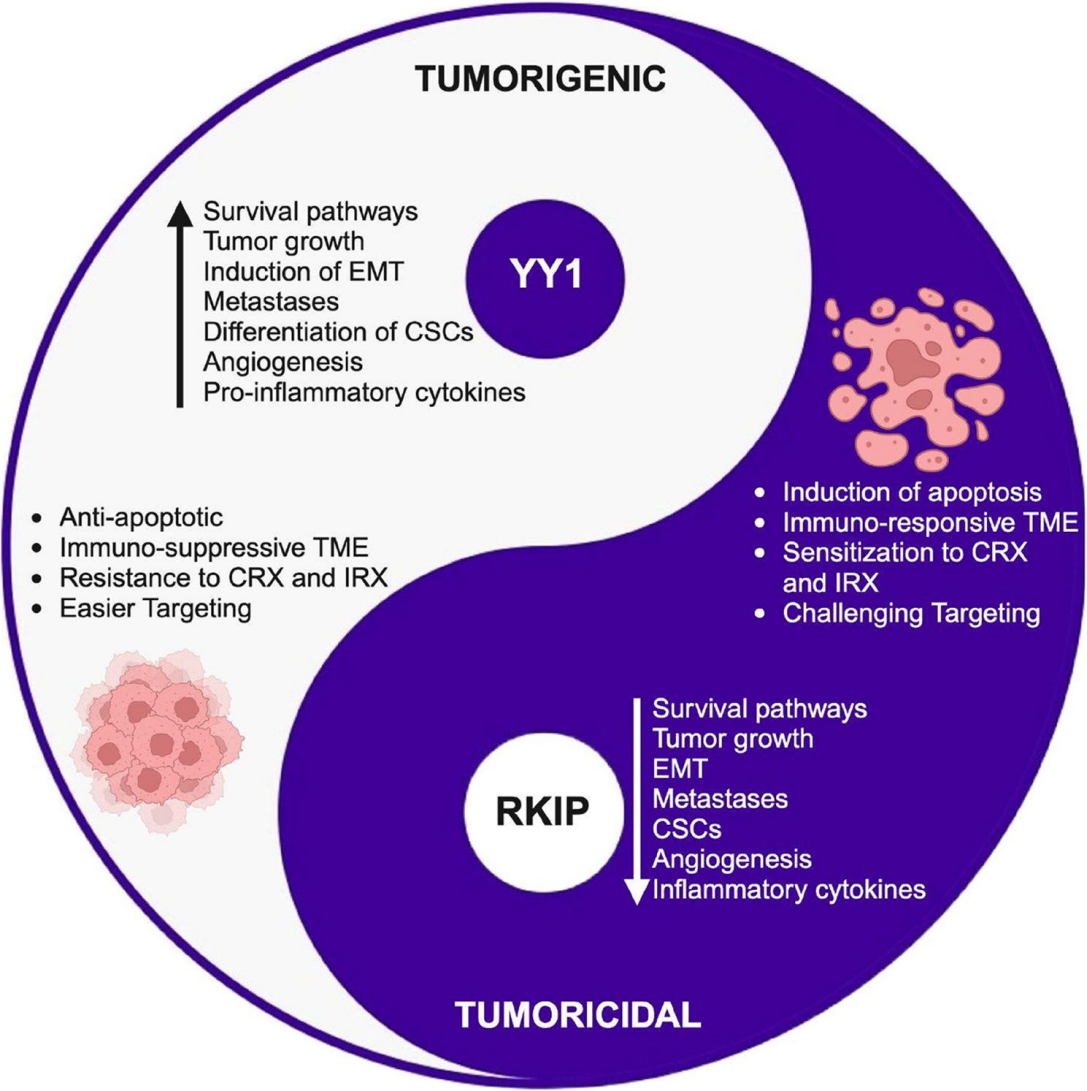

## Highlights

YYR represents a bidirectional regulatory framework: YY1 represses RKIP, while RKIP.

indirectly modulates YY1 activity through inhibition of YY1-driven pathways.

RKIP is a tumor suppressor usually under expressed in cancer.

A dysregulated YYR mediates the pathogenesis and immune evasion in cancer.

A new therapeutic modality is to target the inhibition of YY1 and the induction of RKIP.

## Introduction

Cancer is a highly complex disease, encompassing a wide range of histologically and genetically distinct types that affect various tissues and organs. Despite their differences, many cancers share common features, such as genetic mutations that drive the activation of oncogenes or the loss of tumor suppressor genes. These mutations disrupt fundamental cellular processes, including proliferation, invasion, metastasis, and mechanisms of drug resistance. Additionally, cancer progression can be influenced by hormones, metabolic changes, and immune evasion mechanisms [1–4]. Over the years, multiple waves of cancer treatments have emerged, including chemotherapy targeting DNA replication, drugs inhibiting survival signaling pathways, monoclonal antibodies targeting tumor-specific receptors, and immunotherapy aimed at restoring immune system function [5–7]. While these therapies have significantly improved patients outcomes, many cancers exhibit intrinsic or acquired resistance, posing a significant challenge to long-term treatment success.

Recent studies have identified the Raf kinase inhibitor protein (RKIP) and Yin Yang 1 (YY1) as significant players in cancer biology, exerting opposing effects. RKIP is typically downregulated in most cancers [8–10] and functions as a metastasis suppressor [11], immune inducer [12], inhibitor of cancer cell viability and proliferation [13], and a sensitizer to reverse therapeutic resistance [14]. In contrast, YY1 is frequently overexpressed in most cancers [15], acting as an oncogene that promotes epithelial-mesenchymal transition (EMT) and metastasis [16], enhances cancer cell proliferation and survival [17], and sustains resistance to therapies [18].

RKIP exerts its tumor-suppressive effects by inhibiting critical cancer pathways, including Raf/MEK/ERK and NF-κB [8, 19]. Beyond its role in cancer, RKIP is implicated in various physiological processes, such as chromosomal development [20], asthma [21], systemic inflammatory response syndrome [22], Alzheimer’s disease [23], and cardiovascular conditions [24]. Meanwhile, YY1 functions as a transcription factor with context-dependent roles, capable of either promoting or suppressing tumor growth based on its binding interactions [25].

Despite their distinct molecular mechanisms, mounting evidence has revealed an inverse regulatory relationship between RKIP and YY1, underscoring their critical roles in cancer progression, immune modulation, and therapeutic resistance. Given the contrasting and opposing effects of RKIP and YY1 on cancer cells, we have identified the RKIP-YY1 axis as *“YYR”.* In this review, we will be presenting the data on the various molecular vents manifested by RKIP and YY1 and our justification for the new dysregulated YYR(RKIP-YY1) axis in cancer. Targeting this axis, by inducing RKIP or inhibiting YY1, should result in the inhibition of tumor growth and metastases as well as restoring cancer cells’ response to chemotherapeutic and immunotherapeutic interventions.

## Molecular biology of YY1 and RKIP

### YY1: Structure and Function

YY1 is composed of 414 amino acids and contains several key structural domains. The C-terminal domain possesses four GLI-Krüppel-type zinc fingers that are critical for its transcriptional repression function [26]. There are three proposed models on YY1 mediated repression, the first one involving YY1 competitively binding to promoter elements independent of activators functioning on the promoter [27]. Then there are two quenching models that involve activator-specific repression. In type I quenching repression, a protein interacts with the activator itself whereas type II quenching repression interferes with targets of the activator [27]. These models are not mutually exclusive and it is likely YY1 employs multiple mechanisms to achieve repression.

There is additionally an activation domain situated at the N-terminus of YY1 where the adenovirus-derived E1A protein can bind to relieve repression by YY1 [28]. Later research revealed that E1A recruits p300, a transcriptional coactivator, to YY1, facilitating its switch to activation [29]. As an activator, YY1 can compete with inhibitors for binding sites such as when it binds to the Xist 5’ region, preventing repression by REX1 [30]. YY1 also interacts with many co-activators, for example it can enhance the activity of ATF6, a key regulator of endoplasmic reticulum stress [31]. Another significant coactivator of YY1 is INO80, a chromatin remodeling enzyme. The INO80-YY1 complex plays an essential role in gene activation, chromatin accessibility, DNA replication, and DNA repair via homologous recombination [17]. Lastly, post-translational modifications, such as acetylation and deacetylation, influence YY1’s activity. Acetylation by p300 enhances activation, whereas deacetylation by histone deacetylases (HDACs) leads to repression [32].

### RKIP: Structure and Function

RKIP, also known as phosphatidylethanolamine-binding protein 1 (PEBP1), is a multifunctional regulatory protein that integrates signaling across several key pathways. Structurally, RKIP is a 23-kDa globular protein, composed of nine-beta strands and four alpha-helices. There is a hydrophobic ligand-binding pocket formed by two stretches of conserved residues that also serves as a PE-binding domain for phosphatidylethanolamine [33]. Additionally, it binds various ligands at low pH, including O-phosphatidylethanolamine, O-phosphorylserine, O-phosphoryltyrosine, and O-phosphorylthreonine [33]. The ligand-binding pocket of RKIP is particularly important for its interaction with Raf-1. Under normal conditions, RKIP binds Raf-1, but its release is triggered by PKC-mediated phosphorylation at Ser-153, which also increases RKIP’s affinity for GRK2 [24, 34]. Mutations, specifically in the residues that comprise the ligand-binding pocket, have been shown to inhibit RKIP’s inhibitory role highlighting its importance [35].

**Fig. 1:**
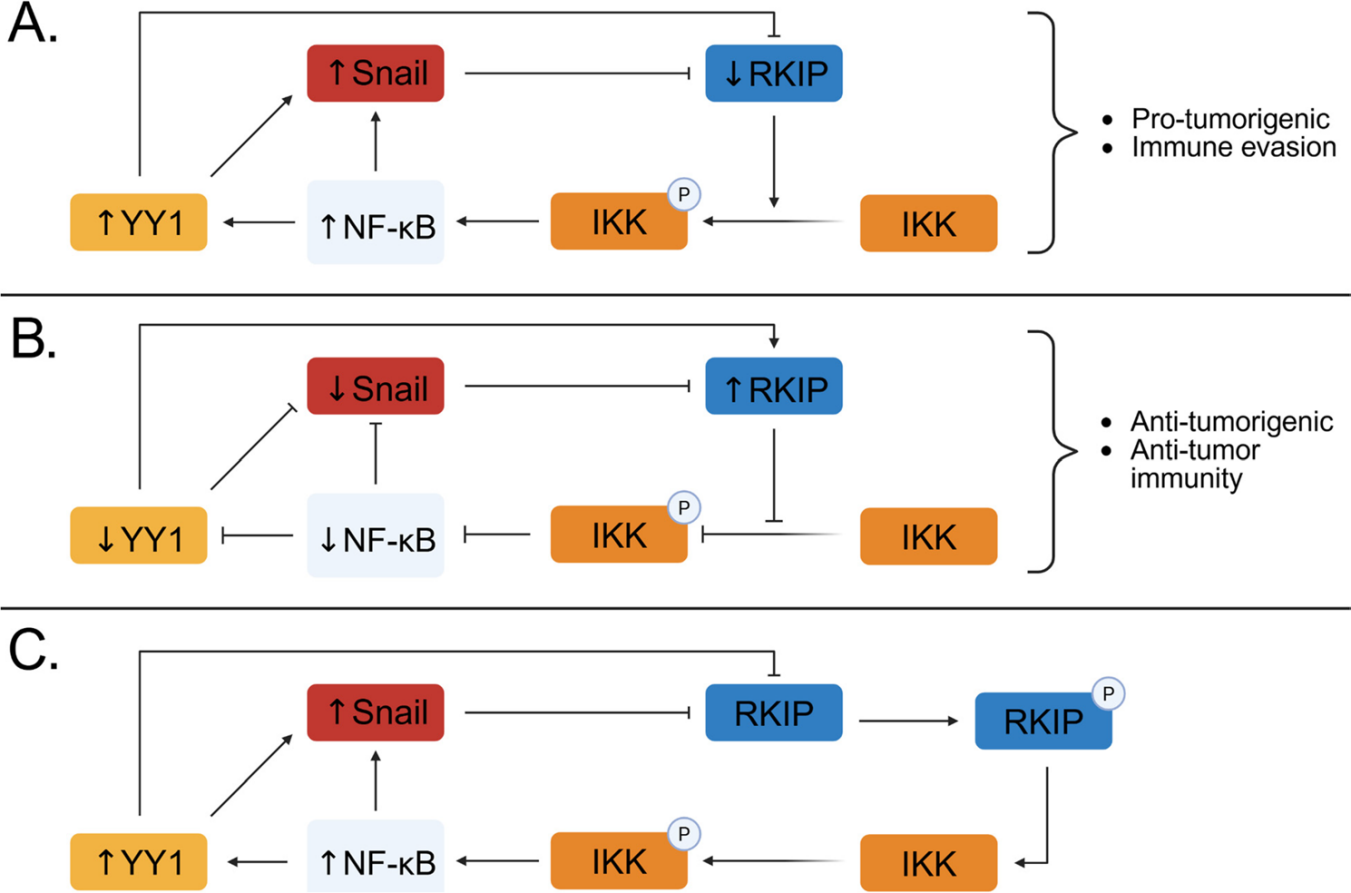
Regulatory feedback loop of YY1 and RKIP. **A** RKIP in its inactive form allows phosphorylation of the IKK complex and subsequent degradation of IκB. This allows activation of NF-κB and upregulation of both YY1 and Snail. As Snail is a transcriptional repressor of RKIP, the expression levels of RKIP are constitutively repressed. **B** RKIP associates with NIK or TAK1 which prevents them from activating the IKK complex. This prevents the degradation of IκB which keeps NF-κB in its inactive form and unable to upregulate YY1 and Snail allowing RKIP to be overexpressed. **C** RKIP in its phosphorylated state is inactive which allows for the upregulation of YY1 in a similar manner to Figure A. The phosphorylation of the IKK complex activates NF-κB and downstream activation of YY1 and Snail. RKIP is repressed by YY1 and Snail as they are continuously upregulated

RKIP primarily exists in two well-characterized states being RKIP^Raf^ and RKIP^GRK2^ which bind to Raf and GRK2 respectively [24]. Recent studies have introduced the presence of an intermediate high-energy state, RKIP^Kin^, which facilitates RKIP phosphorylation and functional switching. Evidence for RKIP^Kin^ comes from experiments involving the RKIP loop mutant P74L, which promotes phosphorylation at S153, enabling the transition from RKIP^Raf^ to RKIP^GRK2^. Although P74 is distant from S153, NMR analysis indicates that P74L increases pocket loop flexibility, stabilizing RKIP^Kin^ and making RKIP more prone to phosphorylation [36]. (Fig. 1)

### The RKIP-YY1 Interaction

The interaction between YY1 and RKIP was reported in the literature as both directly and indirectly. The direct evidence was initially reported by Baritaki et al., 2011 in studies with prostate tumor cell lines that expressed high YY1 and EZH2 and low RKIP expression levels [37]. The authors identified putative binding sites (proximal E-boxes) in the RKIP promoter that bind YY1 and EZH2. Chromatin immunoprecipitation showed that YY1 and EZH2 physically associate with the RKIP promoter. Reporter assays showed that mutation of the E-boxes impacted the ability of YY1 and EZH2 to repress RKIP promoter activity. In addition, silencing YY1 increased RKIP promoter activity, mRNA and protein levels. Conversely, YY1 overexpression correlated with downregulation of RKIP transcription establishing YY1 as a direct negative regulator of RKIP. The detailed mechanisms whereby YY1 recruits co-repressors (e.g. HDACs, EZH2) to the RKIP promoter is better understood in some cancer systems but not in others.

Vivarelli et al., 2022 demonstrated a clear direct regulatory relationship between YY1 and RKIP using the JASPAR prediction tool which identified seven potential YY1 binding sites within the promoter region of RKIP, suggesting that YY1 could directly influence RKIP transcription [38]. This prediction was further supported by analysis of ENCODE ChIP-Seq datasets revealing 23 distinct YY1 binding peaks across nine clusters spanning the RKIP regulatory region. These findings showcase a physical interaction between YY1 and RKIP in both normal and cancer cell models. Moreover, this binding leads to direct transcriptional repression of RKIP expression which was consistently observed in computational datasets where YY1 was upregulated in lung cancer samples and RKIP was downregulated. The authors emphasize that while indirect regulatory mechanisms involving NF-κB and Snail have been proposed in other studies, their data provide direct mechanistic evidence that YY1 itself binds to and represses RKIP expression in lung cancer cells.

Several reports presented indirect evidence of the interaction between YY1 and RKIP. These were discussed in the manuscript with the following paragraph:

In addition to direct repression of RKIP by YY1, YY1 and RKIP engage in an indirect regulatory loop that reinforces cancer progression. YY1 is known to be positively regulated by NF-κB, a transcription factor involved in inflammation, cancer progression, and treatment resistance [37]. RKIP, in turn, is a negative regulator of NF-κB by specifically targeting the NF-κB-inducing kinase (NIK) and Transforming Growth Factor Beta-Activated Kinase 1 (TAK1), which are upstream activators of IκB kinase (IKK). This inhibition ultimately prevents the phosphorylation and degradation of IκB, thereby restricting NF-κB activation and nuclear translocation [39]. YY1 and NF-κB are capable of inducing the expression of the transcription factor Snail, which is a transcriptional repressor of RKIP [40–42]. This creates a feedback mechanism where elevated levels of RKIP will inhibit YY1, NF-κB, and Snail. On the other hand, RKIP in its phosphorylated form is inactive resulting in the overexpression of YY1, NF-κB, and Snail which ultimately contribute to tumor progression, metastasis, and resistance to chemotherapy and immunotherapy [43]. In the above studies, the indirect evidence was correlative or predictive rather than fully demonstrating direct causality.

Overall, the specific binding sites on the RKIP promoter may vary in different cancers. The promoter regions that YY1 binds are not always uniformly mapped across studies. Functional impact in vivo is not studied as cell lines and tissue samples have been examined, in vivo genetic models (e.g. conditional YY1 knockout) investigating RKIP expression are needed.

## Expression of YY1 and RKIP in Various cancers

### Breast cancer

Changes to YY1 expression can have both positive or negative effects depending on the context. In breast cancer, overexpression of YY1 has been associated with decreased Flap Endonuclease 1 (FEN1) expression and increased sensitivity to chemotherapy drugs such as mitomycin C and Taxol. FEN1 plays a crucial role in DNA replication and repair meaning inhibition of YY1 contributes to making cancer cells more resistant to chemotherapy [18]. Another study showed overexpression of YY1 inhibits tumor formation in breast cancer by binding to the promoter of BRCA1 to positively regulate its expression [44]. Further reinforcing the previous findings, YY1 was shown to hinder the growth of human breast carcinoma and glioblastoma cells by inhibiting proliferating cell nuclear antigen (PCNA) expression, a marker of cell growth, and reducing phosphorylation of retinoblastoma protein (pRbSer249/Thr252), which is critical for cell cycle progression [45].

While YY1 can suppress metastasis, several studies have also shown its capability to promote tumor growth. One study identified YY1’s role as a cofactor of activator protein 2 (AP-2) to upregulate the oncogene ERBB2 in breast cancer cell lines. The researchers in this study also noted a significant decrease in ERBB2 protein levels after silencing YY1 using siRNAs [46]. Similarly, it was found that YY1 depletion inhibited clonogenicity, migration, invasion, and tumor growth in breast ductal carcinoma samples. Furthermore, YY1 knockdown altered the architecture of breast cancer cells, making them resemble normal breast epithelial cells, while its overexpression induced malignant features in nontumorigenic cells [47].

Numerous studies have highlighted RKIP’s role as a metastasis suppressor, one study found that RKIP overexpression in breast cancer led to reduced cell adhesion, migration, and invasiveness. On the other hand, mice models injected with RKIP knockdown cells exhibited increased metastasis further emphasizing its role in tumor suppression [48]. Another study by Bach et al. demonstrated RKIP can inhibit metastasis in breast cancer through its inverse relationship with IFN response gene signatures. While IFNs play a crucial role in immune surveillance by enabling the body to recognize and eliminate tumor cells, they can also promote tumor progression by creating a pro-inflammatory tumor microenvironment. The researchers suggest that RKIP suppresses IFN-driven inflammation, thereby reducing the metastatic potential of breast cancer cells [49].

### Prostate cancer

YY1 has been shown to play an essential role in prostate development through its interactions with the androgen receptor (AR) and enhancing transcription of the prostate-specific antigen (PSA) promoter [50, 51]. However, excessive YY1 expression disrupts AR function and YY1 depletion leads to reduced PSA expression, indicating an optimal level of expression necessary for AR transcriptional activity [50]. This is further supported by Huang et al. 2017 where YY1 depletion reduced prostate cancer cell viability, proliferation, and tumor growth while increasing apoptosis, effects that were mediated through the upregulation of miR-146a. Further analyses revealed that YY1 represses miR-146a transcription by binding to its promoter and recruiting EZH2, which contributes to epigenetic silencing [52].

Similar to other cancers, RKIP maintains its role as a tumor suppressor in prostate cancer and its deletion has been shown to contribute to cancer progression. Analysis of RKIP expression using tissue microarrays showcased RKIP expression was significantly reduced in metastatic prostate cancer compared to primary tumors and normal prostate tissue [53]. The loss of RKIP promotes metastasis by increasing the invasive capabilities of prostate cancer cells [9]. In line with this, one study utilized transgenic adenocarcinoma of the mouse prostate (TRAMP) mice, crossbreeding them with RKIP knockout mice to assess the effects of RKIP loss. The findings revealed that RKIP expression declined as prostate cancer progressed, with lower levels observed in metastatic sites compared to primary tumors. Moreover, this study revealed that RKIP deletion does promote metastasis in prostate cancer being associated with elevated phosphorylated ERK and confirming the activation of the MAPK pathway [54]. In a separate study, RKIP overexpression was shown to increase the efficacy of docetaxel, a chemotherapy drug to treat prostate cancer, to ultimately inhibit cell migration and invasion. The researchers of this study noted that upregulating RKIP blocked NFκB’s translocation from the cytoplasm to the nucleus preventing the inactivation of other downstream genes such as E-cadherin, COX, and vimentin [55].

### Hepatocellular carcinoma

YY1 overexpression has been shown to promote tumor growth in hepatocellular carcinoma (HCC). Overexpression of YY1 in HCC cells led to increased cell proliferation and decreased apoptosis even under histone deacetylase inhibitor (HDACi) treatment. On the other hand, YY1 knockdown sensitized cells to the drugs showcasing tumors with significantly reduced growth in response to HDACi [56]. Interestingly, the study also revealed that HDACi treatments themselves downregulated YY1 expression, suggesting a feedback mechanism that could be exploited therapeutically. However, overexpression of HDAC1 increased YY1 levels, emphasizing the interdependent regulation between the two proteins [56]. Similarly, YY1 was found to promote angiogenesis in HCC through the transcriptional activation of vascular endothelial growth factor A (VEGFA). Overexpression of YY1 led to increased VEGFA mRNA and protein levels facilitating angiogenesis by promoting migration, invasion, and tube formation of endothelial cells [57].

RKIP is frequently downregulated in HCC and its loss is closely tied to increased tumor progression. Schuierer et al., 2006 conducted a study on both HCC cell lines and primary human HCC tissues showcasing a negative correlation between RKIP mRNA and tumor progression [58]. Another study showed similar results upon downregulating RKIP levels in cell lines such as HCCLM3 and HepG2 revealing RKIP can suppress cell invasion, motility, and tumor growth [59]. Lastly, in a cohort of 240 HCC patients researchers found RKIP expression to be significantly decreased in tumor tissues compared to adjacent non-tumorous liver. This downregulation was strongly associated with poor differentiation, vascular invasion, lack of tumor encapsulation, and larger tumor size [60].

### Colorectal cancer

Previous studies have established that the transcription factor E2F1 is overexpressed in colorectal cancer (CRC) being involved in the regulation of invasion, migration, and proliferation of cells [61, 62]. Researchers have identified the overexpression of YY1 as a key factor in this pathway which negatively regulates miR-526b-3p and enables E2F1 overexpression. Knockdown experiments confirmed that reducing YY1 expression restored miR-526b-3p levels and decreased E2F1 expression, leading to growth arrest and reduced tumorigenicity in CRC cells [63]. YY1 has also been shown to influence chemotherapy sensitivity, particularly to 5-fluorouracil (5-FU). Silencing YY1 exhibited significantly reduced sensitivity to 5-FU, reduced apoptosis, and led to a strong downregulation of the pro-apoptotic gene BCL2L15. Furthermore, low expression of YY1 and BCL2L15 was associated with metastatic disease and worse relapse-free survival, supporting their tumor-suppressive roles [64]. Lastly, Kim et al., 2021 explored the loss of YY1 in stage III CRC by examining 345 CRC patient samples. YY1 expression was lost in 14.2% of cases, and this loss was significantly associated with larger tumor size, tumor deposits, and higher tumor stage [65].

To explore RKIP’s role in colorectal cancer (CRC), one study used three patient cohorts where the first one involved a broad survey of 279 human tissue samples revealing RKIP is commonly expressed in normal epithelial tissues but reduced in CRC. The second cohort consisted of 268 CRC patients and confirmed that reduced RKIP expression was significantly associated with poorer overall survival. Lastly, the third cohort of 65 early-stage CRC patients helped showcase RKIP expression to be a strong predictor of disease-free survival whereas weak RKIP expression correlated with increased risk of recurrence and shorter disease-free survival [8]. Subsequent studies on this topic held similar results and even delved deeper by examining RKIP expression across different histological zones of CRC [66]. The results showed that RKIP expression was highest in normal mucosa, progressively decreased from the tumor center to the front, and was nearly absent in tumor buds. This gradient of RKIP loss was significantly associated with aggressive tumor features such as distant metastasis, vascular invasion, tumor budding, and infiltrative growth patterns, particularly when loss occurred in the tumor center. Furthermore, tumors with RKIP loss also showed activation of NF-κB and reduced E-cadherin expression, supporting the role of RKIP in suppressing epithelial-mesenchymal transition (EMT), a key step in cancer metastasis [66].

### Multiple myeloma

In multiple myeloma (MM) and patient-derived bone marrow samples, YY1 levels are much higher in comparison to normal B-cells. Further analysis of 30 MM bone marrow samples and 5 normal controls using IHC and flow cytometry confirmed that both nuclear and cytoplasmic YY1 expression were markedly elevated in MM patient cells [67]. Using microarray datasets and gene set enrichment analysis, a strong correlation between YY1 overexpression and nonhyperdiploid MM was identified which is a subtype with a poorer prognosis and frequent immunoglobulin H translocations [68, 69]. In addition to MM progression, YY1 can influence chemoresistance where researchers used siRNA to silence YY1 in MM cell lines. This knockdown significantly sensitized cells to bortezomib- and melphalan-induced apoptosis, demonstrating YY1’s involvement in chemoresistance. This study also confirmed that YY1 mRNA levels were elevated in MM compared to normal plasma cells and monoclonal gammopathy of undetermined significance (MGUS). Notably, YY1 expression increased with clinical stage severity, further reinforcing its relevance in MM pathogenesis [70].

Typically in cancers RKIP is downregulated however, multiple myeloma (MM) demonstrates an unusual overexpression of RKIP, primarily in its inactive phosphorylated form (pRKIP). Shvartsur et al., 2017 conducted a bioinformatic analysis using Oncomine datasets to compare gene expression profiles between pre-malignant MM and MM samples. They found that genes like Bcl-2, DR5, PTEN, and TNF-α were overexpressed in MM, while Bcl-6, Fas, and TNFR2 were underexpressed. The researchers suggest the overexpression of inactive pRKIP in MM may contribute to the sustained activation of survival pathways, resulting in increased expression of oncogenes like Bcl-2 and decreased expression of pro-apoptotic genes like Fas [43].

### Melanoma

Melanoma has been shown to have a high susceptibility to YY1 deregulation as heterozygous loss of YY1 significantly reduced tumor burden and increased survival. Upon YY1 deletion it was found that both oxygen consumption and protein synthesis rate decreased showcasing its role in maintaining metabolic needs of melanoma cells [71]. In relation to this, one study established YY1 is not only essential for primary melanoma formation, but its loss also promotes metastasis. Through gene expression analysis following YY1 knockdown in melanoma cell lines, the study found that YY1 positively regulates genes involved in cell cycle and proliferation, while repressing genes linked to cell adhesion, epithelial-to-mesenchymal transition (EMT), and TGF-β signaling [72]. The researchers of this study suggest YY1 knockdown induces a “phenotype switching” where melanoma cells transition from a proliferative state to an invasive state [73].

RKIP acts as a potent tumor suppressor in melanoma by dampening key signaling pathways and its expression is consistently downregulated in metastatic melanoma cells. One study found an inverse correlation with the expression of Melanoma differentiation associated gene-9 (MDA-9), a known driver of melanoma metastasis, and RKIP [74]. Elevated levels of MDA-9 can inhibit RKIP via ERK-mediated activation of Snail whereas elevated levels of RKIP can physically interact with MDA-9 to reduce its expression and disrupt pro-metastatic downstream signaling. Moreover, RKIP expression has been associated with improved immune responsiveness in a study with melanoma patients receiving dendritic-cell vaccine immunotherapy [75]. Post-vaccination the data showed elevated levels of RKIP correlated with increased survival and enhanced T-cell responses while negatively correlating with markers of myeloid inflammation.

### Cervical cancer

Several studies have investigated the abnormal expression of YY1 in cervical carcinoma and how it contributes to tumor progression. Wang et al. utilized immunohistochemical analyses of cervical cancer at various stages demonstrating significantly higher YY1 expression in cervical squamous cell carcinomas compared to normal cervical tissue. In addition, elevated YY1 correlates with advanced FIGO stage and inversely correlates with E-cadherin, supporting a role in promoting EMT and invasion [76]. Another study found the transcription factor drives the expression of plasminogen activator urokinase (PLAU), a protease implicated in cell migration and invasion. YY1 activates PLAU by binding to its promoter which in turn promotes MMP-9 and cyclin D1 transcription while suppressing E-cadherin [77].

In an analysis of 210 patients’ cervical tissues, it was found that RKIP was consistently downregulated in cervical cancer patients with lymph node metastases. Notably, this was not observed in those without lymph node metastases supporting RKIP’s role as a metastasis inhibitor [78]. Martinoho et al. findings agree with the previously mentioned study and showed RKIP can promote aggressive cancer behaviors. After analyzing 259 cervical tissue samples the researchers of this study noted RKIP is significantly more expressed in benign lesions compared to cervical cancer. Utilizing a BrdU assay, there was an increase in the amount of RKIP-deficient cells in the G2/M + S phase and decrease in G0/G1 phase. In addition, low RKIP expression did not confer greater tumor size, however there was a notable increase in blood vessel recruitment suggesting a role in tumor vascularization. Lastly, the absence of RKIP was associated with resistance against the chemotherapy drug cisplatin [79].

### Gastric cancer

Emerging evidence has shown that YY1 plays a critical role in the progression of gastric cancer as one study revealed YY1 expression was upregulated in gastric cancer cell lines and primary gastric cancers [80]. Moreover, elevated YY1 nuclear expression correlated with reduced survival rate, deeper tumor infiltration, and lymph node metastasis [80, 81]. Upon siRNA-mediated knockdown of YY1 in multiple gastric cancer cell lines there was a marked inhibition of proliferation, monolayer colony formation, G1 cell cycle arrest, and increased apoptosis [80]. Similar results were seen in a separate study that demonstrated YY1 facilitates the advancement of gastric cancer using the CCDC43/ADRM1 signaling axis. CCDC43 (Coiled-Coil Domain Containing 43) physically interacts with ADRM1 (Adhesion-Regulating Molecule 1) to stabilize its protein and enhance the ubiquitin-proteasome pathway that supports tumorigenesis. YY1 is able to bind and activate both CCDC43 and ADRM1 allowing for their overexpression which was associated with poor differentiation, lymph node metastasis, and reduced overall survival [82].

Utilizing immunohistochemistry and western blot techniques, gastric cancer samples were analyzed to assess RKIP levels. RKIP was significantly downregulated and was associated with poorer tumor differentiation, greater depth of invasion, and the presence of lymph node metastasis [83, 84]. To further investigate RKIP’s functional role, the researchers overexpressed RKIP in the human gastric cancer cell line MKN45 using a eukaryotic expression vector. The overexpression of RKIP significantly reduced the cells’ invasiveness and metastatic potential in vitro, as demonstrated through a Transwell invasion assay [83]. Another study investigated the role of RKIP in gastric cardia adenocarcinoma (GCA), a small region where the stomach meets the esophagus [85]. Hypermethylation of RKIP’s promoter region was found to contribute to its decreased expression in GCA and resulting in higher recurrence of cancer, presence of vascular invasion, and worse survival rate [86].

## Cross-talk signaling pathways

As previously discussed, one of the main pathways that highlights the reciprocal role between YY1 and RKIP is the NF-κΒ/Snail/YY1/RKIP signaling loop. YY1 facilitates promoting tumor cell survival and resistance to apoptosis by suppressing the expression of death receptors, Fas and DR5, on anti-tumor CD8 T cells [87–89]. In contrast, RKIP functions as a tumor suppressor and apoptosis sensitizer by inhibiting the NF-κB signaling cascade. As RKIP expression is upregulated, this results in the inhibition of NF-κB activity which subsequently downregulates YY1 transcription [90]. Mechanistically, overexpression of RKIP not only downregulates YY1 transcription but derepresses YY1-suppressors of death receptors and enhances tumor cell responsiveness to TRAIL-mediated apoptosis [88]. Within the NF-κB/Snail/YY1/RKIP loop, hyperactivation of NF-κB promotes YY1 and Snail while suppressing RKIP and creates a self-reinforcing circuit that maintains a resistant and invasive tumor phenotype.

**Fig. 2:**
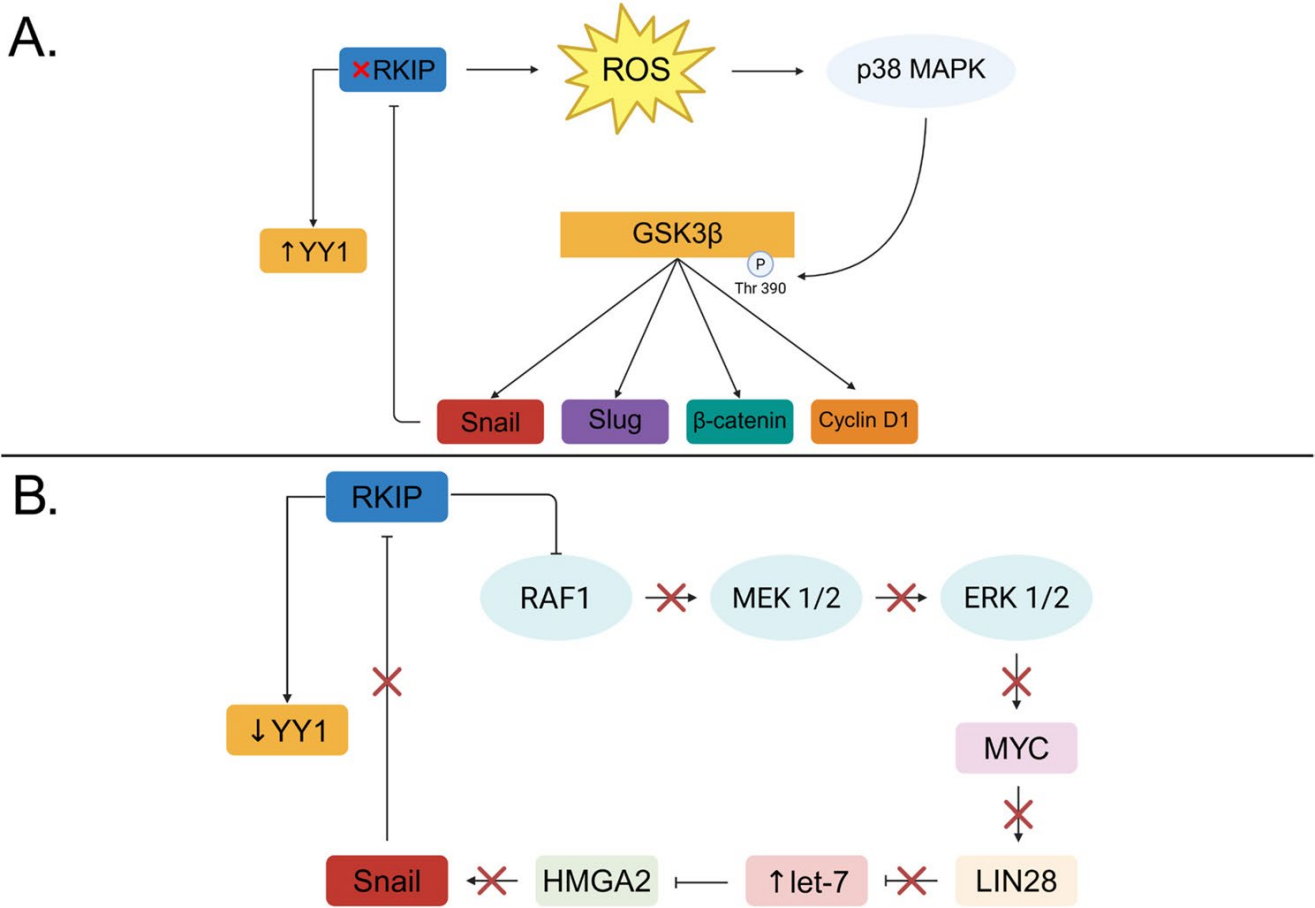
Cross-talk signaling pathways. **A** RKIP deficient cells generate elevated amounts of ROS which are potent activators of p38 MAPK. In its active form, p38 MAPK phosphorylates GSK3β and upregulates β-catenin, Snail, Slug, and cyclin D1. Elevated Snail expression inhibits RKIP via the NF-κB pathway which increases YY1 levels. **B** RKIP disrupts the RAF/MEK/ERK signaling cascade which prevents Myc from enhancing Lin28 expression Inhibition of Lin28 alleviates its repression over the miRNA let-7 and subsequently inhibits HMGA2, an activator of Snail. Downregulation of HMGA2 also decreases Snail allowing for overexpression of RKIP followed by YY1 suppression

Glycogen synthase kinase 3 (GSK3) is a serine/threonine kinase that plays a role in various biological processes including cellular transport, protein synthesis, cell signaling, and apoptosis [91, 92]. RKIP has been shown to regulate this protein, specifically GSK3β, as protein levels were significantly reduced in RKIP knockout cells [93]. Mechanistically, loss of RKIP increases the amount of reactive oxygen species which facilitates the activation of p38 MAPK [94]. The activated kinase phosphorylates GSK3β at threonine 390 which is an inhibitory site that dysregulates GSK3β while upregulating protein levels of β-catenin, Snail, Slug, and cyclin D1 [95]. Not only will this promote EMT, but elevated levels of Snail will inhibit RKIP and upregulate YY1 via the NF-κB pathway [40, 96]. On the other hand, cells overexpressing RKIP will stabilize GSK3β and prevent the accumulation of β-catenin, Snail, Slug, and cyclin D1. The lower levels of Snail will derepress RKIP and allow it to inhibit YY1. (Fig. 2A)

Previous studies have highlighted RKIPs role in regulating let-7, a miRNA that plays a role in both tumor growth and metastasis [97]. To start off this signaling cascade, RKIP inhibits Myc via the Raf/MEK/ERK pathway [98]. Myc binds to the promoter of LIN28 and enhances its transcription, but in RKIP-expressing cells, decreased MAPK signaling leads to reduced Myc activity, thereby lowering LIN28 levels [99]. When LIN28 is downregulated by RKIP, it disrupts its role as an inhibitor of let-7 allowing this miRNA to accumulate. One major target of let-7 is HMGA2, a chromatin remodeling factor that promotes the expression of Snail [100, 101]. Therefore, elevated levels of let-7 prevents HMGA2 from inducing Snail and in turn derepress RKIP allowing for its overexpression and NF-κB-mediated YY1 inhibition [40, 96]. (Fig. 2B)

Another crosstalk pathway has been elucidated in prostate cancer where YY1 interacts with androgen receptors (AR) to regulate its expression [50]. YY1 directly binds to the C-terminal domain of AR and enhances AR-driven transcription of target genes such as prostate-specific antigen (PSA). Moderate overexpression of YY1 enhanced AR activity however, excessive YY1 expression inhibited AR activity [50]. Thus, optimal levels of YY1 can induce RKIP expression as previous studies have identified RKIP as a target gene of androgens and AR. Furthermore, excessive or lack of YY1 expression interferes with AR activity and disrupts the AR-mediated transcriptional modulation of RKIP [102].

At the level of signaling pathways, YY1 exerts its oncogenic influence by activating tumor promoting pathways while simultaneously inhibiting tumor suppressive pathways. Through analysis of reverse phase protein array data it was found that YY1 has a strong inducing effect on the cell cycle, DNA damage, and apoptosis resistance [103–106]. Additionally, YY1 has an inhibitory effect on the RAS/MAPK pathway and epithelial-to-mesenchymal transition (EMT) depending on the cancer context [16, 37]. On the other hand, RKIP serves as a potent inhibitor of several metastatic pathways such as the Raf-1/MEK/ERK signaling cascade, a key pathway involved with cell proliferation, differentiation, and survival [98]. Normally Raf-1 is activated by the initial activation of Ras which leads to the phosphorylation of MEK and activation of ERK. Activated ERK translocates to the nucleus and regulates gene expression by phosphorylating transcription factors [107]. RKIP comes into play by selectively binding to either the N-region of the Raf-1 kinases domain or dissociating the Raf-1/MEK complex [108]. The presence of RKIP has been shown to block the downstream activation of the MAPK pathway and prevent cancer progression [109]. Moreover, RKIP negatively regulates autophagy by directly binding to the LC3 protein, interfering with autophagosome formation, and stimulating the AKT/mTORC1 pathway, further curbing survival mechanisms that tumor cells use under stress conditions​ [110]. Thus, while YY1 actively drives tumor growth through oncogenic signaling, RKIP counters these effects by silencing survival pathways and limiting autophagic support.

## Contrasting Roles of YY1 and RKIP in the Pathogenesis of Cancer

### Angiogenesis and tumor microenvironment

The production of novel blood vessels known as angiogenesis is essential to the survival of tumors as they facilitate the transportation of nutrients, oxygen, metabolites, and waste [111, 112]. In response to tumor microenvironments that lack the necessary oxygen or nutrients to thrive induce the production of angiogenic factors, namely vascular endothelial growth factor A (VEGFA) [113, 114]. Previous studies have established YY1’s role in regulating VEGF in multiple types of cancers either directly or indirectly. In vitro studies of hepatocellular carcinoma found a positive correlation between YY1 and VEGFA. Mechanistically, YY1 binds to the promoter of VEGFA increasing its transcriptional activity and facilitates the phosphorylation of VEGFR2 which is needed to promote angiogenesis [115]. Another study shows YY1 can help form new blood vessels by forming a complex with HIF-1α to bind and activate VEGFA, VEGFB, and VEGFC [116]. As opposed to the direct ways YY1 can drive neovascularization, studies on acute myelogenous leukemia (AML) have highlighted indirect methods as well. YY1 was found to be overexpressed in AML cells and binds directly to the small nucleolar RNA host gene 5 (SNHG5) promoter as confirmed through ChIP and luciferase reporter assays [117]. SNHG5 is a long noncoding RNA that acts as a competing endogenous RNA for miR-26b and prevents it from inhibiting connective tissue growth factor (CTGF) to ultimately promote the expression of VEGFA [118].

While the presence of YY1 can help form new blood vessels, the loss of RKIP has been shown to do the same. Fu et al. first explored this by overexpressing RKIP in prostate cancer cells showing decreased vascular invasion which suggest it may possess anti-angiogenic properties [9]. Previous studies on RKIP have shown it may influence angiogenesis through its inhibitory role on tumor associated macrophages (TAMs) recruitment. TAMs play a critical role in maintaining the tumor microenvironment as they polarize into either M1-TAMs with anti-tumor properties or M2-TAMs with pro-tumor properties, depending on the cytokines and growth factors present [119–121]. RKIP can promote TAMs specialization into M1 by inhibiting CCL5, a pro-inflammatory cytokine. In vivo studies showcased tumors expressing RKIP exhibited significantly less vascularization as indicated by a reduction in infiltrating macrophages and CD31 positive endothelial cells in the primary tumors [42, 122]. RKIP likely exerts its regulatory control over CCL5 through the IKK-NF-kB axis as this was previously shown to regulate the expression of multiple chemokines in lung cancer cell lines [123]. Although this exact mechanism in breast cancer cells remains to be fully elucidated, the findings support the idea that RKIP modulates the tumor microenvironment by interfering with pro-inflammatory and pro-angiogenic signaling.

### Immune infiltration in the TME

YY1 and RKIP both modulate immune and inflammatory processes within the tumor microenvironment, but do so in opposing manners. Immune infiltration analyses revealed YY1 correlates positively with immunosuppressive cell types including M2 macrophages and regulatory T cells [124]. This suggests YY1 may contribute to an immunosuppressive tumor microenvironment as seen in breast cancer models where naive CD4 + T cells were found to convert to regulatory T cells supporting immunosuppression [125]. Conversely, YY1 expression was negatively correlated with immune-reactive cells such as CD8 + T cells, follicular helper T cells, and memory B cells which are all critical for promoting anti-tumor immunity through cytokine secretion and enhancing T cell activation. The mechanisms involved that allow YY1 to evade clearance by immune cells is through immune checkpoint activation. YY1 has been shown to enhance the expression of immune inhibitory receptors such as PD-1 and CTLA-4 [126, 127]. Moreover, YY1 can upregulate programmed cell death ligand 1 (PD-L1) by inhibiting the expression of miR-34a or PTEN via p53 [128, 129]. By increasing the expression PD-L1 allows for increased binding to PD-1 and suppresses T-cell mediated immune responses [130]. Beyond its role in T cells, YY1 also influences cancer-associated fibroblasts by enhancing their recruitment and activation through modulation of TGF-β and PDGF signaling pathways [131]. In urothelial bladder cancer cells, CAFs drive the production of miR-146–5p which can further amplify YY1 expression and promote the activation of cancer stem cell markers such as SOX2, KLF4, ALDH1A1, NANOG, or OCT4 [131, 132]. These interactions not only promote tumor aggressiveness but also create a stromal environment that impedes immune cell infiltration and sustains immune suppression. Lastly, YY1 facilitates immune escape through inhibition of antigen presentation by either directly or indirectly repressing MHC class I and II expression, thereby impairing T cell recognition [133, 134].

**Fig. 3:**
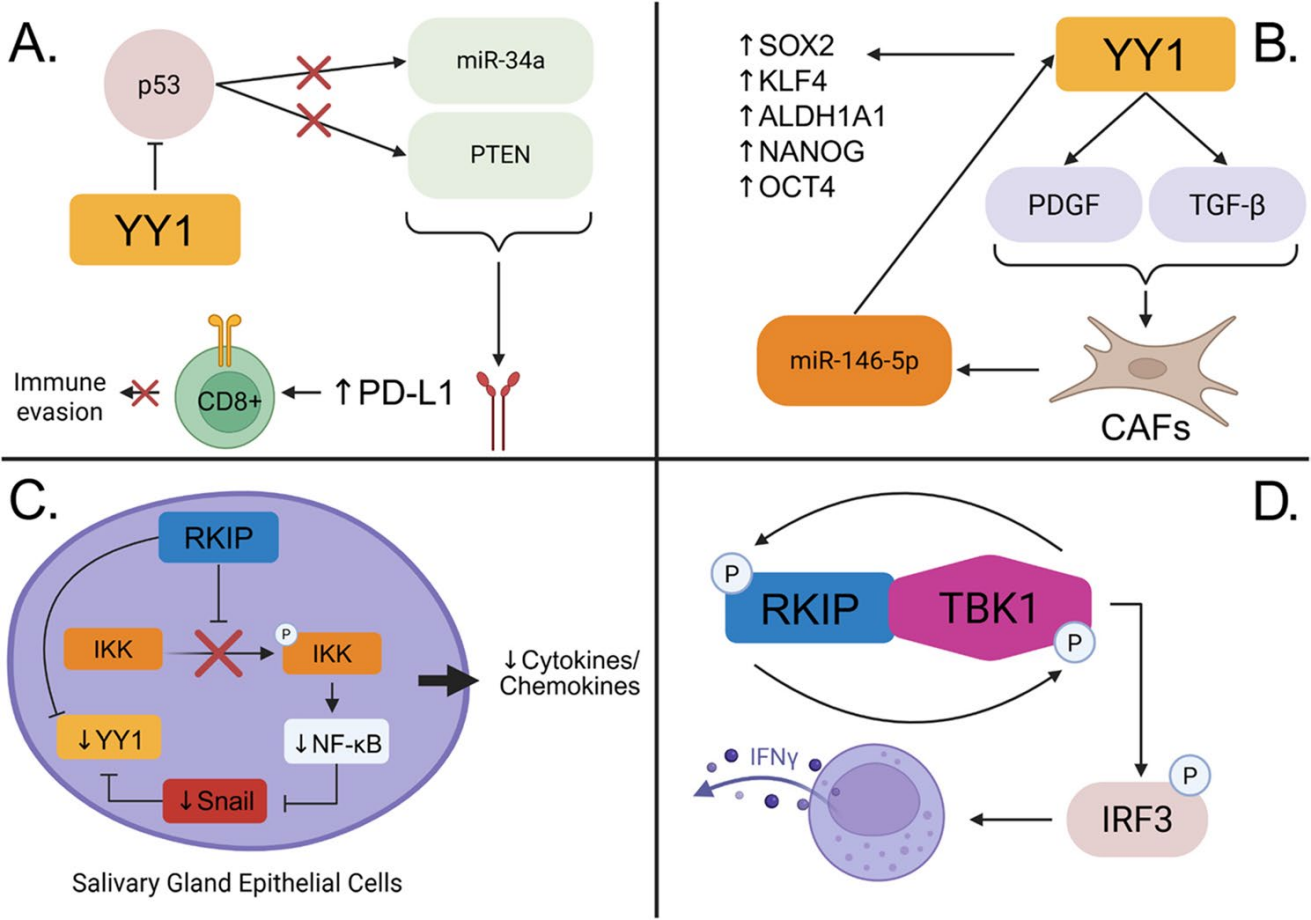
Immune evasion strategies.** A** YY1 inhibits p53 preventing it from activating miR-34a and PTEN, both of which normally repress PD-L1. Uplifting the block on PD-L1 transcription allows for increased binding with PD-1 and suppression of T-cell mediated immune responses. **B** Cancer associated fibroblasts (CAFs) induce the production of miR-146-5p to amplify YY1 expression and promote the expression of stem cell markers such as SOX2, KLF4, ALDH1A1, NANOG, and OCT4. As YY1 levels are elevated, a feedback loop is created where YY1 activates TGF-β and PDGF signaling pathways to further enhance CAFs levels. **C** Overexpression of RKIP prevents phosphorylation of IKK resulting in the downregulation of NF-κB. Specifically in Sjögren’s syndrome, the reduced levels of NF-κB facilitate a reduction in cytokines such as IL-6, IL-8, or TNF-α. **D** RKIP and TBK1 engage in a positive feedback loop by phosphorylating each other at specific residues. Upregulation of TBK1 contributes to a pro-inflammatory tumor microenvironment as it plays a pivotal role in phosphorylating IRF3, a transcription factor that is responsible for triggering the release of type I interferons

For RKIP, higher expression was associated with reduced infiltration of regulatory T cells and memory CD4 + T cells while positively correlated with mast and NK cells which are both involved with anti-tumor responses [124]. This suggests that RKIP plays a role in forming a favorable immune landscape which can be attributed to its involvement with inflammatory responses within the body. As it has been established that RKIP is a negative regulator of NF-κB signalling, one study showed that RKIP deletion significantly activated the NF-κB pathway and induced inflammatory cytokines and chemokines to be released in primary Sjögren syndrome [135]. In addition, a study on systemic inflammatory response syndrome demonstrated RKIPs ability to regulate type I/II interferon production as RKIP deficient cells generate a significantly diminished interferon gamma (IFNγ) response by CD8 + T cells compared to wild type [22]. Similarly, RKIP induces type I interferon production by forming a positive feedback loop with TANK-binding kinase 1 (TBK1), a kinase that activates interferon regulatory factor 3 (IRF3) necessary to generate pro-inflammatory cytokines [136]. This loop begins with TBK1 enhancing its binding affinity with RKIP by phosphorylating RKIP at serine 109. Once bound, RKIP promotes TBK1 autophosphorylation at serine 172 enhancing TBK1s activation. Aside from cytokine regulation, RKIP also influences T-cell function as higher expressions of RKIP were correlated with effective T-cell gene signatures and decreased expressions of inflammatory markers such as MAPK1, Notch1, and STAT3 [75, 137]. In gastric cardia adenocarcinoma, low RKIP expression is linked to diminished T cell function and increased lymph node metastasis [138]. Overall, RKIP and YY1 operate to influence immune function but through inverse mechanisms. RKIP predominantly suppresses inflammation and tumor immune evasion whereas YY1 often promotes immunosuppression. (Fig. 3)

### Cell cycle

Previous studies have highlighted the downregulation of RKIP contributing to cell proliferation and migration in hepatocellular carcinoma via the ERK/MAPK signaling cascade [139]. Al-Mulla et al., 2010 found consistent results that revealed RKIP downregulation not only promoted cellular proliferation, but additionally attributed it to enhanced cell cycle progression [140]. Cell cycle analyses showed that RKIP silencing particularly accelerated G1/S and G2/M transitions as demonstrated by the reduced time from nuclear envelope breakdown to anaphase and quicker completion of the S-phase. Furthermore, RKIP depleted cells upregulated the expression of cyclin D1, D2, E2, cdc6, MCM 2, 4, 6, 7, cdc45L, and SKP2 which are genes that promote G1/S transition [141, 142]. As for G2/M regulators, RKIP downregulation was associated with promoting genes vital for mitotic progression including APC11, APC7, and NEK6 [143, 144]. Lastly, downregulation of aurora B, cyclin G1, and SIRT2, which are known to enforce G2/M checkpoints and maintain chromosomal integrity suggest RKIP loss allows cells to bypass mitotic surveillance [145–147].

In contrast to the pro-proliferative effects of RKIP loss, downregulation of YY1 impairs DNA repair and cell cycle progression. In human pancreatic β-cells, YY1 was found to bind to genomic regions associated with pathways critical for chromatid cohesion, DNA damage recognition, and both G1/S and G2/M checkpoints [148]. To further confirm the importance of YY1, mouse models with YY1 silenced showed reduced β-cell mass, increased DNA damage, and higher β-cell apoptosis. The authors of this study suggest YY1 deletion fails to activate DNA damage response pathways due to the decreased expression of key β-cell identity genes including Ins1, Glut2, Nkxy6.1, and PDX1. Another study also shows YY1 can act as a direct substrate for aurora B by phosphorylating serine 184 within the transcription factors central glycine/alanine rich regulatory domain [149]. Given that RKIP negatively regulates Aurora B expression, this raises the possibility of a functional crosstalk between RKIP and YY1, potentially linking RKIP’s influence on mitotic progression with YY1-mediated transcriptional control.

### Epithelial-mesenchymal transition (EMT)

RKIP works in conjunction with a variety of EMT related proteins such as Snail, which hold a mutual inhibition loop between each other. Snail directly represses RKIP by binding to E-box elements within its promoter while RKIP inhibits Snail by blocking the MEK/ERK signaling cascade that activates it [40, 57]. Overexpression of Snail represses E-cadherin expression and drives the induction of EMT, whereas RKIP functions as an inhibitor of Snail, thereby counteracting EMT and preserving the epithelial phenotype [150, 151]. Moreover, RKIP has been shown to influence another EMT biomarker vimentin through its ability to suppress the Notch1 signaling pathway. Under hypoxic conditions Notch1 is activated and promotes the production of EMT proteins such as vimentin, however RKIP prevents this by cleaving the Notch intracellular domain [152]. Lastly, various cancer models have highlighted the loss of RKIP leads to the upregulation of the mesenchymal adhesion molecule N-cadherin. This occurs through RKIPs inhibitory effects on pro-EMT signaling pathways such as MEK/ERK and thereby indirectly inhibits Snail and N-cadherin expression [153].

While RKIP tends to have an inhibitory effect on EMT, YY1 has been shown to promote EMT. Considering the previously mentioned NF-κΒ/Snail/YY1/RKIP signaling cascade, YY1 promotes the expression of Snail which suppresses epithelial markers and induces mesenchymal markers [154]. One marker is E-cadherin which is inhibited as Snail is overexpressed resulting in increased cell motility and accelerating the EMT phenotype [150, 151]. On the other hand, various studies have shown that Snail can promote a mesenchymal phenotype by upregulating N-cadherin, vimentin, and fibronectin which all contribute to increased cell invasiveness and migration [155–157]. In addition, a study on hepatocellular carcinoma has shown that YY1 can form a transcriptional complex with p65 and p300 to activate the quaking (QKI) gene. The overexpression of QKI leads to downregulating E-cadherin while upregulating vimentin. In addition, QKI promotes the formation of circular RNAs such as miR-615-5p and miR-381-3p which modulate downstream EMT-related genes [158].

### Tumor suppressors

RKIP has been shown to be involved with various tumor suppressors contributing to its well established role as a metastasis suppressor and apoptosis inducer in cancer. One major mechanism involves RKIPs regulation of the Myc/Lin28/Let-7 signaling cascade, leading to the upregulation of let-7 miRNAs and subsequent downregulation of pro-metastatic factors such as HMGA2 and BACH1 [159–161]. These transcription factors are both critical drivers of tumor progression and metastasis, primarily through the promotion of EMT, suppression of apoptosis, and modulation of key signaling pathways [162, 163]. In addition, RKIP can indirectly modulate tumor suppressors such as the p53/p21Cip1/Rb axis by inhibiting the Raf/MEK/ERK signaling cascade, which is required to suppress this axis by blocking p53 acetylation at K161/162 [164].

Similarly, YY1 has been shown to regulate various tumor suppressors depending on the type of cancer involved. Lee et al., 2011 showed that overexpression of YY1 in breast cancer was positively correlated with the tumor suppressor BRCA1 and inhibited cell proliferation [44]. Importantly, these inhibitory effects were dependent on BRCA1 as BRCA1 knockout cells did not demonstrate anti-tumor characteristics. Studies on pancreatic cancer have found overexpression of YY1 promoted Bax expression levels which is a pro-apoptotic member of the Bcl-2 family [165]. Bax plays an important role in removing damaged cells as it helps increase membrane permeability and release of other pro-apoptotic factors into the cytoplasm [166].Given the dual nature of this transcription factor, YY1 can also negatively regulate tumor suppressors such as FEN1, a multifunctional nuclease involved in DNA replication and repair [18]. YY1 has also been shown to downregulate p53 by disrupting its interaction with the coactivator p300, which normally acetylates and stabilizes p53. Moreover, YY1 facilitates the formation of the p53-Mdm2 complex enhancing Mdm2-mediated ubiquitination and degradation of p53 [104].

### Check point inhibitors

Utilizing several mechanisms, RKIP regulates multiple immune checkpoint molecules, playing a significant role in enhancing cancer immunotherapy efficacy. One mechanism involves the MAPK pathway which RKIP inhibits. A study on oral squamous cell carcinoma showed the Jak2-Stat3/MAPK-AP1 pathway regulates PD-L1 supporting an inverse relationship with RKIP [167]. In addition, cytokines like IL-1β and IFN-γ also contribute to PD-L1 upregulation via MAPK signaling [168]. RKIPs inhibitory effects with this signaling pathway further supports its negative regulation on PD-L1 expression. Similarly, strong inverse correlations were identified between RKIP and several other immune checkpoint molecules including PD-L2, BTLA, CD96, TIGIT, and CSF1R in prostate cancer samples [169]. These findings were consistent across both primary and metastatic prostate cancer datasets, suggesting that high RKIP expression is associated with reduced immune evasion and a less immunosuppressive tumor microenvironment.

Through various layers of regulation, YY1 modulates PD-L1 to contribute to a more immunosuppressive tumor microenvironment unlike RKIP [170]. Transcriptionally, it directly binds to the CD274 promoter which is the gene encoding PD-L1 and modulates signaling pathways such as IFN-γ/JAK/STAT, PI3K/Akt/mTOR, and IL-6/STAT3 to enhance PD-L1 transcription [171]. Epigenetically, YY1 interacts with histone deacetylases (HDACs) and enhancer of zeste homolog 2 (EZH2), contributing to chromatin remodeling and PD-L1 expression [172, 173]. Post-transcriptionally, YY1 suppresses miR-34a and miR-200 which are both negative regulators of PD-L1 through inhibition of p53 and direct interaction with miRNA promoter regions [128, 171]. Aside from PD-L1, Balkhi et al., 2018 revealed that YY1 directly binds to the promoters of PD1 and LAG-3, enhancing their transcription. Moreover, Tim3 lacks a YY1 binding site but its expression can be indirectly upregulated by YY1 through GATA3 which can interact with YY1 to enhance transcriptional activity [126].

### Resistance to cell death by apoptosis

As a tumor suppressor, RKIP can promote apoptosis in multiple ways including the inhibition of pro-survival signaling pathways such as the RAF/MAPK cascade. Evident by its name, RKIP blocks RAF1’s kinase activity by dissociating the RAF1/MEK complex and inhibiting RAF1’s activation by binding to its N-region [19, 35, 54]. This disrupts RAF-1 mediated signaling with various pro-apoptotic factors such as ASK1 or BAD [174, 175]. In addition to suppressing RAF1 kinase, RKIP also interferes with NF-κB signalling by inhibiting upstream kinases such as TAK1, NIK, and IKKα/β [39]. NF-κB is often constitutively active leading to increased expression of cIAPs (cellular inhibitors of apoptosis), c-Flip Cellular FLICE (FADD-like IL-1β-converting enzyme)-inhibitory protein, and BCL2 family proteins which all play important roles to inhibit apoptotic pathways [176, 177]. Inhibition of these key survival pathways by RKIP have been shown to facilitate restoration of apoptosis resistant cells whereas knocking down RKIP expression in sensitive cells conferred resistance [42, 178].

In opposition to RKIP, YY1 commonly functions as a suppressor of apoptosis in cancer by upregulating anti-apoptotic genes. Bcl-2 and Bcl-xL are key anti-apoptotic members of the Bcl-2 protein family that regulate cell death and are directly correlated with YY1 expression [179]. Mechanistically, this involves the NF-κB/YY1/Snail/RKIP/p53 signaling loop where YY1 represses p53 which would normally downregulate Bcl-2 and Bcl-xL [180]. Alternatively, YY1 stabilizes Snail which inhibits RKIP and activates Bcl-2 and Bcl-xL downstream [181]. Either way the upregulation of these Bcl-2 proteins via increased YY1 expression contributes to apoptosis as they inhibit mitochondrial outer membrane permeabilization leading to the release of cytochrome c and subsequent activation of caspases [182]. Another protein YY1 modulates is survivin, a member of the inhibitor of apoptosis (IAP) family that regulates both mitosis and apoptosis by forming chromosomal passenger complexes and blocking caspase activation [183, 184]. YY1’s role in survivin regulation is context dependent as it can activate or repress its transcription depending on the cofactors present [185].

### Cellular energetics

Utilizing a more indirect approach, RKIP has been shown to modulate cellular energetics through its interactions with the miRNA let-7. As previously mentioned, RKIP inhibits let-7 targets such as HMGA2 and BACH1 which have been shown to cooperate with each other to significantly advance tumor progression in breast cancer studies [186]. The inhibition of BACH1 specifically prevents it from repressing genes in the electron transport chain thus supporting mitochondrial oxidative metabolism [187]. In addition, RKIP has been shown to reduce the transcription of LIN28 which would normally promote aerobic glycolysis and suppress mitochondrial oxidative phosphorylation, further promoting tumor progression [161]. Pinheiro, 2021 investigated how RKIP regulates cellular metabolism in non-small lung cancer cell lines. Through in silico analysis, RKIP expression correlates positively with genes involved in oxidative phosphorylation and negatively with glycolysis related genes. Moreover, upon RKIP overexpression glycolytic enzymes like HK2 and LDHA were downregulated suggesting RKIP shifts the tumor metabolic phenotype away from the Warburg effect [188]. Beyond cancer, RKIP has been found to negatively regulate glucose induced cellular processes in human retinal capillary endothelial cells (HRCECs). Overexpression of RKIP suppressed glucose induced cell viability, migration, angiogenesis, and EMT demonstrating its essential role in cellular energetics [189].

YY1 has been shown to modulate cellular metabolism, particularly aerobic glycolysis, through regulation of lactate dehydrogenase A (LDHA). Wang et al., 2022 showed that YY1 is significantly overexpressed in neuroblastoma cell lines resulting in elevated glucose uptake and lactate production. Conversely, knockdown of YY1 reduced aerobic glycolysis which was further confirmed by the decreased expression of key glycolytic genes including GLUT1, HK2, PDHK1, and LDHA [190]. In another study on neuroblastoma, YY1 was shown to facilitate aerobic glycolysis by binding to the promoter of MZF1, a zinc-finger transcription factor that upregulates glycolytic genes such as HK2 and PGK1 [191]. When YY1 expression is high, MZF1 is transactivated, leading to enhanced glucose uptake, lactate production, ATP generation, and increased tumor cell viability and invasiveness [191]. Similarly, in glioblastoma YY1 was found to promote glycolysis and tumor progression through transcriptional activation of G protein subunit Gamma 5 (GNG5). Liang et al., 2024 demonstrated that YY1 directly binds to the GNG5 promoter correlating with poor prognosis, whereas YY1 suppressed cell proliferation and glycolytic activity [192]. Overall, RKIP supports mitochondrial oxidative metabolism and suppresses glycolysis while YY1 directly enhances glycolytic activity highlighting their opposing influences on cellular energetics.

## Therapeutic Implications of Targeting the YYR Axis

### Targeting YY1

Multiple studies have investigated the role of YY1 in cancer therapy as dysregulation of its transcription holds significant promise due to its role in tumor progression, therapy resistance, and microenvironment regulation. One major avenue involves the use of small molecule inhibitors such as DETA-NONOate. As a nitric oxide donor, DETA-NONOate can inhibit YY1 through S-nitrosylation, thereby inhibiting its DNA binding ability and sensitizing tumor cells to Fas-induced apoptosis [70]. Similarly, RRx-001 is also a nitric oxide donor that induces cytotoxicity under hypoxic conditions by inhibiting the IKK complex, an upstream regulator of NF-κB and YY1 [193]. In addition, a study on breast cancer tissue revealed another small molecule that can directly regulate YY1, betulinic acid. Downregulation of YY1 subsequently reduced the expression of HER2 and its downstream kinases further inhibiting tumor progression [194]. Mechanistically, betulinic acid binds to cannabinoid receptors which disrupts the constitutive repression of ZBTB10, a transcriptional repressor of YY1, by miR-27a [194]. Lastly, a more targeted approach utilizes JAC1 (JWA Activating Compound 1) which binds directly to YY1 and relieves its repression over the ARL6IP5 gene. Restoration of this gene allows it to carry out its various tumor suppressive functions such as inducing apoptosis and chemoresistance reversal [195, 196].

Peptide-based strategies offer another promising route to regulate YY1 with YY1BM (YY1-blocking micropeptide) being a notable example. This micropeptide is encoded by LINC00278 and binds to YY1 which disrupts its interaction with the androgen receptor, followed by eEF2K downregulation. eEF2K is a useful therapeutic target as it has been identified as an indicator of stomach adenocarcinoma and decreased expression of eEF2K in esophageal squamous cell carcinoma facilitated greater apoptosis [197, 198]. Synthetic peptides mimicking YY1’s oncoprotein binding (OPB) domain have also been designed to disrupt YY1’s interactions with partners like EZH2. Blocking the recruitment of EZH2 upregulated the tumor suppressors PTENP1 and PTEN contributing to the reduction in breast cancer cell proliferation and tumor growth seen in xenograft mouse models [172]. Lastly, a study on prostate cancer demonstrated that YY1 is highly expressed in M2-polarized tumor associated macrophages, where it promotes tumor progression by upregulating IL-6 expression [199]. This regulation is mediated through YY1’s ability to form nuclear condensates via liquid-liquid phase separation (LLPS) in M2 macrophages, enabling interactions with the IL-6 enhancer and promoter to increase its expression [199]. Knockdown of YY1 in M2 macrophages has been accomplished through a polypeptide on a liposome carrier with YY1 siRNA resulting in reduced IL-6 and enhanced CD8 + T-cell infiltration [200].

Rituximab is an anti-CD20 monoclonal antibody that decreases the phosphorylation of the NF-κB pathway resulting in the downregulation of YY1 showcasing an antibody-based therapy that can regulate YY1 [201]. Similarly, galiximab is another antibody that targets CD80 and has been shown to suppress NF-κB signaling, inhibit AKT phosphorylation, and ultimately downregulate YY1 [179].

Researchers have also explored modulating YY1 levels through the use of miRNAs which target YY1’s 3’ UTR and prevent translation. miR-34c is an example that functions as a negative regulator of myoblast proliferation and a promoter of differentiation by directly targeting YY1 [202]. Another study on colon cancer used miR-7 to directly inhibit YY1 resulting in decreased cell proliferation [203]. Extensive work on the use of various other miRNAs including miR-381, miR-29a, or miR-584-3p have been previously investigated highlighting its promise as a therapeutic agent [81, 204, 205].

Gene editing technologies, particularly CRISPR/Cas9, represent a frontier in YY1-targeted therapy. Liu et al., 2022 found that YY1 is transcriptionally activated by the non-specific lethal (NSL) histone acetyltransferase (HAT) complex in hepatocellular carcinoma [206]. CRISPR/Cas9-mediated knockout of NSL3, a core subunit of this complex, resulted in reduced YY1 expression and impaired cell proliferation [206]. Another study utilized CRISPR/Cas9 to deplete YY1 levels via sgRNAs as YY1 can directly bind and activate phosphofructokinase (PFKP). Upon YY1 inhibition and subsequent decreased expression of PFKP, there was a decrease in tumor cell proliferation and enhanced apoptosis [207] (Table 1).

**Table 1:** YY1 targeting strategies

Therapeutic Agent	Mechanism of Action	References
DETA-NONOate	Nitric oxide donor that inhibits YY1 via S-nitrosylation, suppressing DNA binding and enhancing Fas-mediated apoptosis	[70, 72]
RRx-001	Nitric oxide donor that inhibits IKK and YY1 under hypoxic conditions, increasing cytotoxicity	[193, 194]
Betulinic acid	Suppresses YY1 via cannabinoid receptor signaling that disrupts the miR-27a/ZBTB10 axis	[194, 195]
JAC1	Directly binds YY1 and derepresses ARL6IP5, restoring tumor suppressive activity	[195, 196, 197]
YY1BM	Encoded by LINC00278, disrupts YY1-androgen receptor interaction, downregulates eEF2K	[197, 198, 199]
OPB-domain peptides	Blocks YY1 interaction with EZH2, restore PTENP1 and PTEN, and inhibit tumor growth	[172, 173]
Polypeptide liposome carrier	Uses YY1 siRNA to knockdown YY1 in M2 macrophages resulting in IL-6 reduction and increased CD8 + T cell infiltration	[200, 201]
Rituximab	Anti-CD20 antibody that downregulates YY1 via NF-κB inhibition	[201, 202]
Galiximab	Anti-CD80 antibody that inhibits NF-κB and AKT, reducing YY1 expression	[179, 180]
miR-34c	Targets YY1 3’ UTR to suppress translation, promotes myoblast differentiation	[202, 203]
miR-7	Directly inhibits YY1 expression, reducing colon cancer cell proliferation	[203, 204]
CRISP/Cas9 (NSL3 knockout)	Reduces YY1 expression by targeting upstream HAT complex	[206, 207]
CRISPR/Cas9 (YY1 sgRNAs)	Directly targets YY1 or its binding site at PFKP, reducing proliferation	[207, 208]

### Targeting RKIP

Considering RKIP is usually downregulated in most cancers, therapeutic strategies involving restoration or mimicking RKIP function holds significant promise due to its tumor-suppressive roles in metastasis, immune modulation, and apoptosis. Synthetic drugs such as didymin, dihydroartemisinin, or rituximab upregulate RKIPs expression leading to increased apoptosis due to the inhibition of the ERK1/2 and NF-κB pathways [201, 208, 209]. Another drug, epirubicin, was found to enhance RKIP in breast cancer models by acting through NME1 (non-metastatic cells 1) which is a transcriptional activator of RKIP leading to reduced cell migratory capabilities [210].

Some other therapeutic strategies for inducing RKIP expression involve targeting its key repressors such as Snail, BACH1, and EZH2. A small molecule, CYD19, binds to Snail and disrupts its interaction with CBP/p300 resulting in Snail’s degradation and its inability to repress RKIP [211]. BACH1 is another RKIP repressor that may be targeted using hemin which has been shown to reduce metastasis [212, 213]. Lastly, EZH2 inhibition can be accomplished in various ways to ultimately induce RKIP such as the use of methyltransferase inhibitors [214]. Another approach aims to promote EZH2 degradation through the use of GNA002 or the long non-coding RNA ANCR. Collectively, these approaches aim to relieve RKIP repression, thereby restoring its tumor-suppressive functions and enhancing cancer treatment efficacy [215, 216].

The use of long non-coding RNAs (lncRNAs) have been shown to regulate RKIP with lncRNA XIST being a notable example. In a study on prostate cancer, XIST was found to be significantly downregulated correlating with advanced tumor stage, poor prognosis, and increased metastasis [217]. Mechanistically, XIST functions as a competing endogenous RNA by sponging miR-23a which directly targets the 3’ UTR of RKIP mRNA for degradation. Overexpression of XIST alleviates the inhibition of RKIP by sequestering miR-23a resulting in a reduction in cell growth and inducing G0/G1 cell cycle arrest [217]. This XIST/miR‑23a/RKIP axis represents a compelling therapeutic target, and future approaches may involve RNA-based therapies to upregulate XIST or inhibit miR-23a.

Epigenetic silencing through promoter methylation or histone deacetylation is a well documented mechanism for RKIP downregulation which makes epigenetic therapies a good target for RKIP regulation. In various gastrointestinal and breast cancers, methylated RKIP promoters are correlated with low expression and poor clinical outcomes [86, 218, 219]. Previous studies have shown an increase in RKIP mRNA and protein through the use of 5-azacytidine, a demethylating agent, suggesting further exploration with other agents [220, 221]. Another epigenetic pathway involves histone modifications, specifically deacetylation which have been implicated in RKIP repression. A study on prostate cancer cell lines used the HDAC inhibitor trichostatin A resulting in an upregulation of RKIP expression [40]. Similar findings were seen in triple-negative breast cancer (TNBC) cell line SUM159 however, other TNBC cell lines demonstrated compensatory upregulation of BACH1 negating the benefits which suggests the use of combination therapies (Table 2).

**Table 2:** RKIP targeting strategies

Therapeutic Agent	Mechanism of Action	References
Epirubicin	Enhances RKIP expression through NME1 activation, reducing cell migration in breast cancer	[210, 211]
CYD19	Disrupts Snail interaction with CBP/p300, degrading Snail and lifting RKIP repression	[211, 212]
Hemin	Targets BACH1, a repressor of RKIP, to suppress metastasis	[212, 213, 214]
EZH2 Inhibitors (GNA002, lncRNA ANCR)	Targets EZH2, a repressor of RKIP, to inhibit its transcription and restore RKIP expression	[215, 216, 217]
lncRNA XIST	Sponges miR-23a to prevent RKIP mRNA degradation and induce cell cycle arrest	[217, 218]
5-Azacytidine	DNA methyltransferase inhibitor that demethylates RKIP promoter	[220, 221, 222]
Trichostatin A	HDAC inhibitor that increases RKIP expression through histone acetylation	[40, 42]

## Discussion

The present findings underscore a critical role of the established dysregulated YYR axis in promoting immune evasion, therapeutic resistance, and tumor aggressiveness across multiple cancer types. The overexpression of YY1, a multifunctional transcription factor, correlates with downregulation of RKIP, a well-established tumor suppressor. The inverse relationship between YY1 and RKIP expression is consistent with our and others published reports indicating that YY1 directly and indirectly represses RKIP transcription.

This repression facilitates activation of downstream oncogenic signaling cascades, particularly the NF-κB, PI3K/Akt and MAPK/ERK pathways, all of which are known to modulate immune responses and contribute to the upregulation of immune checkpoint molecules, such as PD-L1 or CTLA-4. YY1-mediated RKIP suppression leads to enhanced expression of immunosuppressive molecules and remodeling of the TME, shifting it in favor of immune escape. Conversely, RKIP serves as a molecular checkpoint that not only inhibits pro-tumorigenic signaling but also maintains immune surveillance by preserving antigen presentation, pro-inflammatory cytokine signaling and the activation of anti-tumor CD8 cytotoxic T cells. Hence, loss of RKIP disrupts this balance, reinforcing its role in facilitating effective anti-tumor immunity and maintaining an immune-reactive TME.

The clinical relevance of this dysregulated YYR axis is further supported by the observation that high YY1 and low RKIP expression profiles are associated with poor prognosis [223, 224], increased metastasis [82, 225](Wang et al., 2017; Wang et al., 2020), and resistance to immune checkpoint blockade (ICB) [126, 226] in patient cohorts. These findings suggest that the YY1-RKIP axis may serve as a biomarker for stratifying patients likely to respond poorly to immunotherapy and as a therapeutic target to overcome resistance.

We presented several approaches to disrupt the YYR axis that offer potentially efficacious therapeutic interventions to overcome immune evasion. promising avenues. Various agents were described to inhibit YY1 and restore RKIP expression, including small molecules, peptides, RNA-based therapeutics, and gene editing tools that re-sensitize tumors to immunotherapy, and suppress tumor-promoting signaling pathways. Conversely, we also presented various means for the direct restoration of RKIP via demethylating agents, HDAC inhibitors, or suppression of its key repressors that similarly restore immune recognition and impede immune evasion. The integration of such approaches with existing immunotherapies, such as PD-1/PD-L1 blockade therapies, may produce synergistic effects and expand the spectrum of responsive patients.

## Challenges and Future Perspectives

Despite growing evidence supporting the pathological relevance and therapeutic potential of the YYR axis in cancer, several questions that must be answered remain. While numerous studies have demonstrated an inverse correlation between YY1 and RKIP expression, the mechanistic underpinnings of their interaction remain incompletely defined. Further exploration is necessary to determine whether this regulatory axis is consistent across other cancer types, particularly regarding the interplay with tumor mutational burden, epigenetic regulation, and the microbiome. Additionally, in vivo validation across diverse tumor models will be critical to confirm the causal role of this axis in immune evasion and its potential for therapeutic intervention. While preclinical studies have identified promising compounds that modulate YY1 and RKIP, issues related to specificity or bioavailability remain significant barriers.

## Conclusion

The YY1-RKIP cross talk represents more than a simple antagonistic interaction- it is a regulatory axis central to cancer progression and therapeutic response. The proposed nomenclature YYR serves to crystallize this concept. It provides a scaffold for future investigations into the complex dynamics of transcriptional and signaling networks in oncology. It also represents a novel strategy to target YYR for effective response to therapies.

## Data Availability

No datasets were generated or analysed during the current study.

## Abbreviations

YY1: Yin Yang 1
RKIP: Raf Kinase Inhibitory Protein
NF: κB-Nuclear Factor kappa-light-chain-enhancer of activated B cells
EMT: Epithelial-Mesenchymal Transition
MAPK: Mitogen-Activated Protein Kinase
ERK: Extracellular signal-regulated Kinase
HDAC: Histone Deacetylase
EZH2: Enhancer of Zeste Homolog 2
TME: Tumor Microenvironment
PD: L1-Programmed Death-Ligand 1
CAF: Cancer Associated Fibroblast
AR: Androgen Receptor
CRISPR: Clustered Regularly Interspaced Short Palindromic Repeats
BACH1: BTB and CNC homolog 1
VEGFA: Vascular Endothelial Growth Factor A
HMGA2: High Mobility Group A2
LDHA: Lactate dehydrogenase A
STAT3: Signal Transducer and Activator of Transcription 3
